# Decellularized bovine jugular vein and hand-sewn ePTFE valved conduit for right ventricular outflow tract reconstruction in children undergoing Ross procedure

**DOI:** 10.3389/fcvm.2022.956301

**Published:** 2022-09-07

**Authors:** Haoyong Yuan, Ting Lu, Zhongshi Wu, Yifeng Yang, Jinlan Chen, Qin Wu, Sijie Wu, Hong Zhang, Tao Qian, Can Huang

**Affiliations:** ^1^Department of Cardiovascular Surgery, The Second Xiangya Hospital of Central South University, Changsha, China; ^2^Engineering Laboratory of Hunan Province for Cardiovascular Biomaterials, Changsha, China

**Keywords:** Ross procedure, pediatric, DP-Bovine Jugular Vein, Hand-sewn ePTFE valved conduit, freedom from graft failure

## Abstract

**Background:**

The Ross procedure is recommended as an optimal aortic valve replacement (AVR) in children and young adults due to several advantages. Nevertheless, multiple reconstructions of the right ventricular outflow tract (RVOT) with new valve conduits have caused some concern regarding the durability of the Ross AVR. Decellularized bovine jugular vein conduit (BJVC) (DP-BJVC) and hand-sewn expanded polytetrafluoroethylene valved conduits (ePTFE VC) are widely employed to reconstruct the RVOT with satisfactory long-term outcomes. However, few studies have compared the safety and efficacy between the two valve conduits. We aimed to evaluate the early outcomes and report our single center experience in the application of these conduits.

**Methods:**

Twenty-two pediatric patients (aged < 18 years) who underwent Ross procedures with DP-BJVC and ePTFE VC in our center between 1 June, 2017 and 31 January, 2022 were enrolled. The Kaplan–Meier method was used to evaluate survival, freedom from RVOT reintervention, and freedom from RVOT graft dysfunction. Mixed-effects analysis with the Geisser–Greenhouse correction and Sidak's multiple comparisons test for *post-hoc* analysis was employed to compare the peak gradient across the conduit at varying follow-ups.

**Results:**

All patients were followed up in full. The total early survival rate was 90.9%; two patients in the DP-BJVC group died. There was no significant difference in early mortality, cross-clamp time (*p* = 0.212), in-hospital stay (*p* = 0.469), and RVOT graft thrombosis or endocarditis between the two groups. There was similarly no significant difference between Kaplan–Meier freedom from RVOT graft dysfunction curve (*P* = 0.131). The transprosthetic gradient gradually increased over time in both groups and was significantly higher in the DP-BJVC group at follow-up (*P* < 0.05).

**Conclusions:**

Both conduits show excellent early and midterm outcomes for RVOT reconstruction in the Ross procedure. We suggest that DP-BJVC is more suitable for infants, and ePTFE conduit is more suitable for older children who require larger conduits.

## Introduction

The Ross procedure is recommended as an optimal aortic valve replacement (AVR) in children and young adults, due to advantages such as the long time survival without anticoagulation therapy, excellent hemodynamics and exercise ability, and growth potential of the pulmonary valve autograft (1). Nevertheless, multiple reconstruction of the right ventricular outflow tract (RVOT) with new valve conduit and neo-aortic root dilatation may turn a single-valve disease into a double-valve pathology requiring reoperation, which causes some concern regarding the durability of the Ross AVR (2). Due to limited availability and high costs of pulmonary allografts (AGs), regarded as the golden standard for RVOT, xenografts and artificial material have attracted attention and are considered as alternatives (3).

The Contegra conduit (Medtronic Inc., Minneapolis, MN) is one of the most successful alternatives and is widely used as it has similar hemodynamic performance and durability comparable to Ags (4). Due to unpredictable cytotoxicity and the possibility of early calcification of glutaraldehyde-treated bovine jugular vein conduit (BJVC), and fewer available large size conduits, the rate of RVOT graft dysfunction remains unacceptably high (5). The patient's immune response to residual donor cells and cell debris is considered critically responsible for graft failure (6). Therefore, decellularization technologies have been proposed to reduce conduit immunogenicity and increase durability (7).

Since 2002, the decellularized and photo-oxidatively crosslinking BJVC (DP-BJVC) was employed to reconstruct the RVOT in our hospital, and has achieved satisfactory durability and functionality midterm outcomes in pediatric patients (8). Recently, hand-sewn expanded polytetrafluoroethylene valved conduit (ePTFE VC) is widely employed to reconstruct the RVOT with satisfactory long-term outcomes from Japanese multi-center studies (9). To our best knowledge, few studies have compared the safety and efficacy between DP-BJVC and ePTFE VC.

During the past 4 years, two types of valve conduit have been employed in RVOT reconstructions in Ross patients in our center. In this study, we aimed to evaluate the early outcomes of our cohort and report our single center experience in the application of these conduits.

## Patients and methods

### Patients

We considered all pediatric patients (aged < 18 years) who were to undergo the Ross procedure with DP-BJVC and hand-sewn ePTFE valved conduit (ePTFE VC) at the Second Xiangya Hospital of Central South University in China between 1 June, 2017 and 31 January, 2022. This retrospective study was approved by the Institutional Ethics Committee (reference number: LYF2020097) and written informed consent was obtained from parents or guardians before surgery to allow the use of their data.

### Valve conduit preparation

The DP-BJVC (Yaxin Medical Technology Co., Ltd., Wuhan, China) was produced as previously reported with multi-step decellularization and dye-mediated photooxidation (8). The ePTFE VC was prepared with a Gore-Tex conduit and 0.1 mm ePTFE membrane (WL. Gore and Associates Inc., Flagstaff, AZ). We tailored the ePTFE membrane into three continuous U shapes with the following parameters: W = 3.14 D; H1 = 0.95 D; H2 = 0.65H1, where D was equal to the diameter of the conduit. We then fixed the membrane at the marked position inside the conduit with a 6-0 prolene suture (Ethicon, Inc., Somerville, NJ). We tailored the conduit to the required length after checking the valve function (Supplementary Video 1).

### Surgical technique

A single surgeon performed the Ross procedure in our center and all patients underwent median sternotomy and standard cardiopulmonary bypass with bicaval cannulation under moderate hyperthermia (28–32 °C). A standard root replacement technique with coronary artery reimplantation was employed in the neo-aortic root reconstruction. The RVOT was reconstructed with prepared valve conduits. Conduit size was determined by age and body surface area and converted to z-score based on previously published regression equations (10). Three patients underwent Ross procedures with repair of the associated cardiac abnormalities, including mitral valve replacement (*n* = 2), correction of patent ductus arteriosus, and coarctation of the aorta (*n* = 1). Intraoperative transesophageal echocardiography was used to confirm adequate valve function of the conduit in all cases immediately after the procedure.

Postoperatively, temporary anticoagulation with intravenous heparin was also used during the 24 h after surgery, aimed at an activated partial thromboplastin time (APTT) ratio of 1.5–2.0 times above baseline. Anticoagulation for all conduits was obtained using sodium warfarin, aimed at an international normalized ratio of 1.8–2.0 for one year, and continued with aspirin (3–5 mg/kg, daily).

### Follow-up and data collection

Clinical data were acquired from hospital records and the institute's cardiac database. Transthoracic echocardiography (TTE) was employed to value the stenosis or regurgitation of the valve of conduit after the procedure, and at 1 month, 3 months, 6 months, 12 months, 24 months, 36 months, 48 months, and 60 months. The grade of conduit stenosis was determined using continuous Doppler to measure the maximum velocities across the conduit and the pressure gradient across the RVOT. The grades were as follows: mild, peak velocities <3 m/s and peak gradient <36 mmHg; moderate, peak velocities 3–4 m/s and peak gradient 36–64 mmHg; severe, peak velocities >4 m/s and peak gradient >64 mmHg. The degree of pulmonary regurgitation was classified based on a five-grade semiquantitative scale (0, none; 1, trivial; 2, mild; 3, moderate; or 4, severe) according to the jet flow as measured with pulsed Doppler echocardiography. All the criteria used for grading were based on commonly used guidelines for echocardiograms (11).

### Statistical analysis

IBM Statistical Package for the Social Sciences (IBM SPSS Statistics for Windows, Version 22.0) was used for all data analyses. Descriptive statistical analysis was undertaken using continuous data presented as median and interquartile range (IQR) or mean ± standard deviation after checking the normality, and categorical variables as raw data and/or percentages. The independent samples *t*-test was employed to compare the continuous variables, while Fisher's exact test was performed to compare outcomes for categorical variables. The Kaplan–Meier method was used to evaluate the survival and freedom from RVOT reinterventions, and results were presented with 95% confidence intervals. This was further compared using the Log-rank (Mantel–Cox) test between groups. Mixed-effects analysis with the Geisser–Greenhouse correction and Sidak multiple comparisons test for *post-hoc* analysis was employed to compare the pressure gradient(PG)across the conduit in different follow-up time. *P*-values < 0.05 were considered statistically significant.

## Results

### Demographics

**Figure 1:**
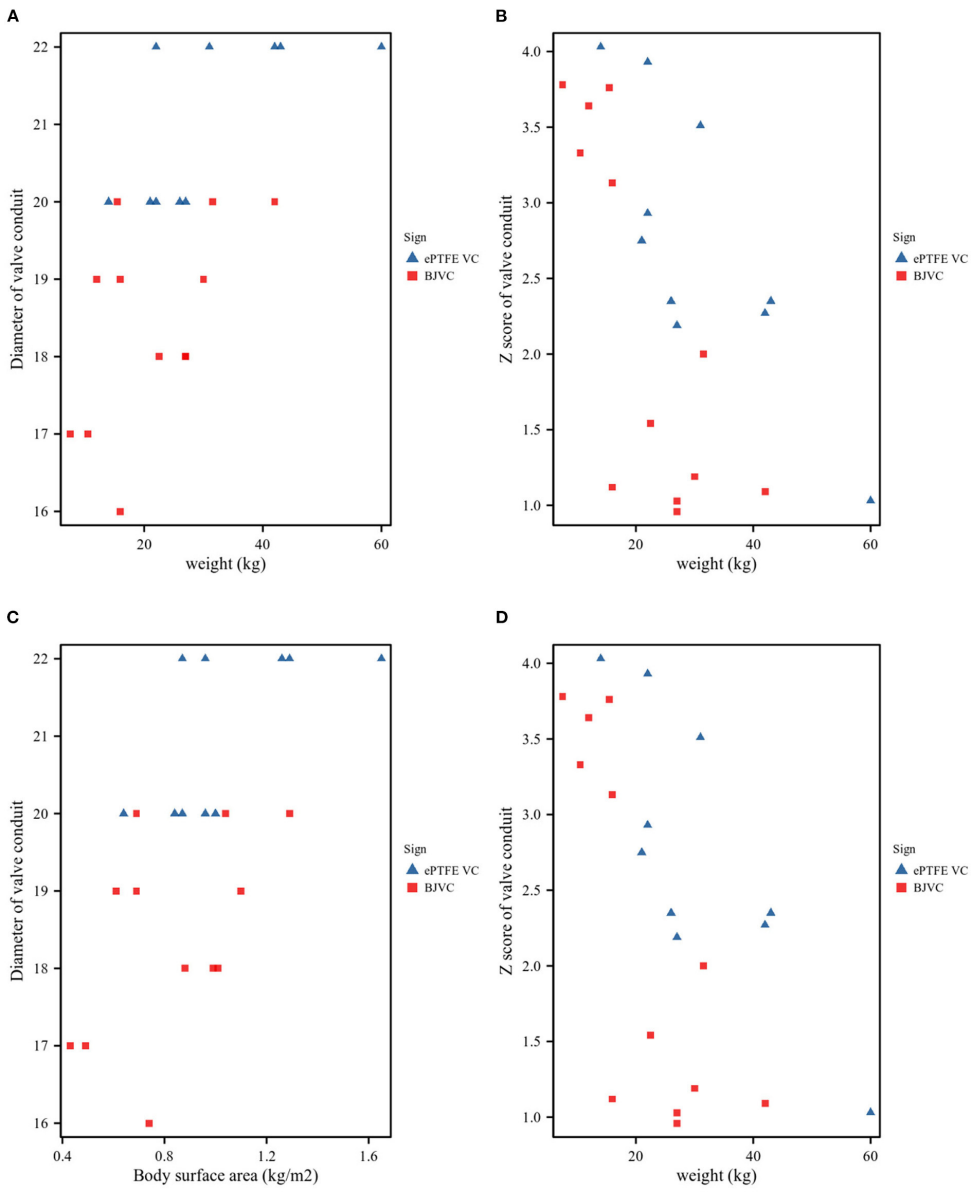
Conduit size. **(A)**. Patient weight and conduit diameters. **(B)**. Patient weight and Z-values of the conduit. **(C)**. Patient body surface area (BSA) and conduit diameters. **(D)**. Patient BSA and Z-values of the conduit.

A total of 22 pediatric patients underwent Ross procedures, and the basic characteristics of the DP-BJVC and ePTFE VC are shown in Table 1. The mean age of the patients treated by DP-BJVC was 6.08 ± 2.93 years, whereas the mean age of the patients treated by ePTFE VC was 8.60 ± 4.01 years. There was no difference in age (p = 0.118); sex (female, *p* = 0.323); body surface area (kg/m^2^, *p* = 0.101); weight (kg, *p* = 0.094); AV hemodynamic lesion, *n* (*p* ≥ 0.056); previous interventions, *n* (*p* > 0.999); LV ejection fraction (mean ± SD, *p* = 0.980); concomitant diseases, *n* (*p* = 0.381); and infective endocarditis, *n* (*p* > 0.999). The diameter of the conduit (mm) was significantly smaller in the DP-BJVC group than that in the ePTFE VC group (*p* < 0.001), whereas no significant difference of the Z-score of conduit was found between the two groups (*p* = 0.266) (Figure 1).

**Table 1:** Basic clinical character of the patients with Ross procedure.

	**Total**	**BJVC**	**ePTFE VC**	***P*-value**
Number of conduits, *n*	22	12	10	
Age (year), mean ± SD	7.23 ± 3.61	6.08 ± 2.93	8.60 ± 4.01	0.118
female, *n*	17	8	9	0.323
Body surface area (kg/m^2^), mean ± SD	0.92 ± 0.29	0.83 ± 0.26	1.04 ± 0.29	0.101
Weight (kg)	25.7 ± 12.59	21.46 ± 10.26	30.8 ± 13.73	0.094
AV haemedynamic lesion, *n*				
Stenosis	14	10	4	0.074
Insufficiency	2	1	1	>0.999
Mixed lesion	6	1	5	0.056
Previous interventions, *n*	3	2	1	>0.999
LV ejection fraction (%), mean ± SD	67.86 ± 5.86	67.83 ± 4.97	67.9 ± 6.92	0.980
Concomitant diseases, *n*	7	5	2	0.381
Infective endocarditis, *n*	5	3	3	>0.999
Diameter of conduit (mm)	19.59 ± 1.76	18.42 ± 1.21	21 ± 1.05	<0.001
Z score of conduit	2.45 ± 1.08	2.22 ± 1.20	2.73 ± 0.91	0.266

### Surgical technique outcomes and early mortality

The total early survival rate was 90.9%, and two patients demised early in the DP-BJVC group: one from damage of the coronary artery, and the other from intracerebral hemorrhage immediately after the operation due to infective endocarditis. There was no obvious difference in early mortality, cross-clamp time (min, *p* = 0.212), in-hospital stay (days, *p* = 0.469), and RVOT graft thrombosis or endocarditis between the two groups (Table 2).

**Table 2:** operative data and follow-up outcomes.

	**ePTFE VC**	**BJVC**	***p* value**
Early motality, *n*	0	2	
Follow-up time(months)	20.10 ± 6.65	29.75 ± 18.31	0.111
Cross-clamp time(min)	148.56 ± 31.52	167.67 ± 35.02	0.212
In-hospital stay(days)	23.75 ± 10.12	28.33 ± 15.98	0.469
RVOT peak gradient ≥50 mmHg	0	3	0.045
RVOT regurgitation ≥moderate	0	1	0.263
RVOT graft reintervention	0	2	0.108
RVOT graft thrombosis or endocarditis	0	0	

### Early follow-up outcomes

**Figure 2:**
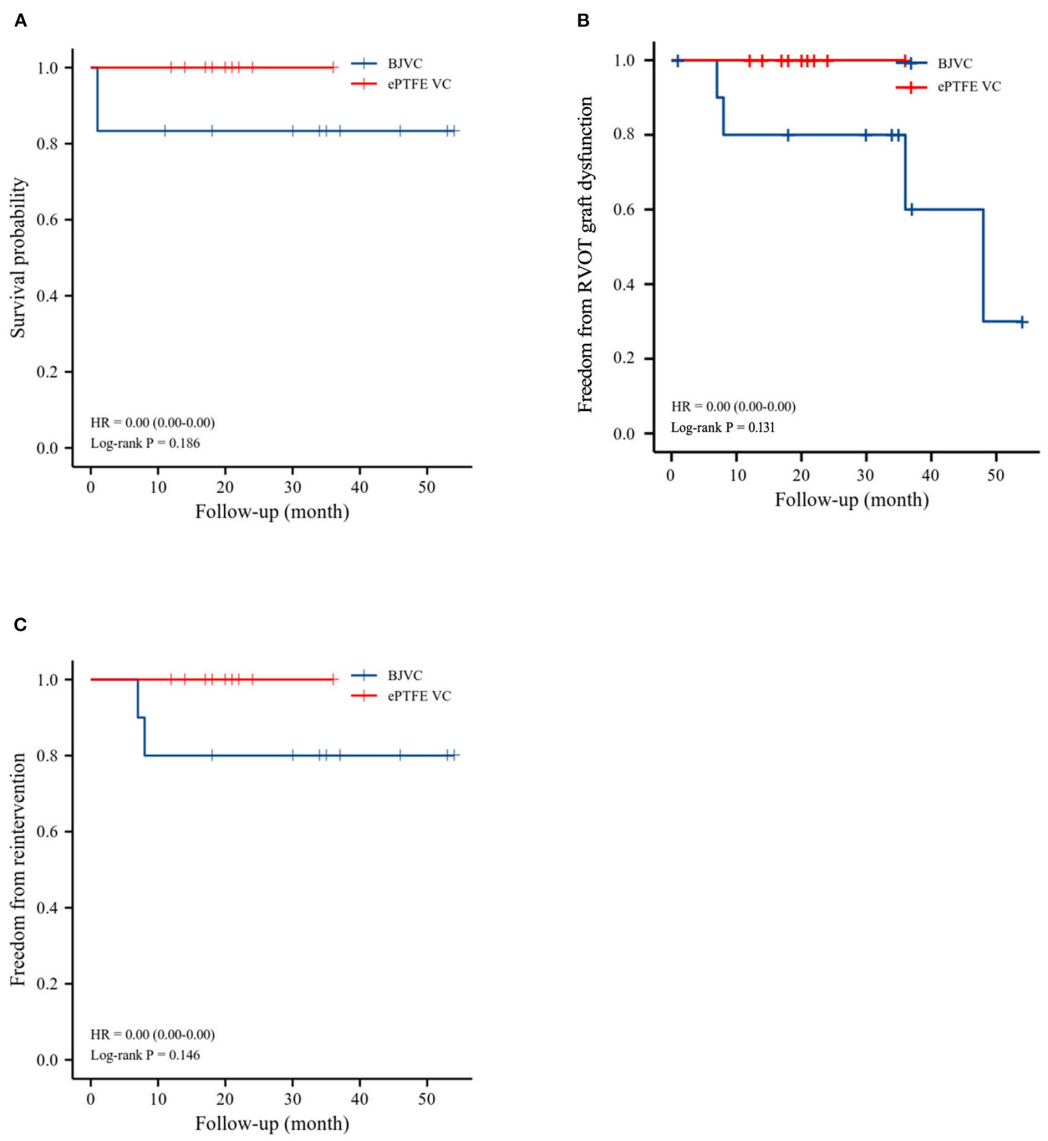
Outcomes of survival, freedom from RVOT reintervention, and freedom from RVOT graft dysfunction, as evaluated by Kaplan–Meier method. **(A)**. Survival. **(B)**. Freedom from RVOT graft dysfunction. **(C)**. Freedom from reoperations.

**Figure 3:**
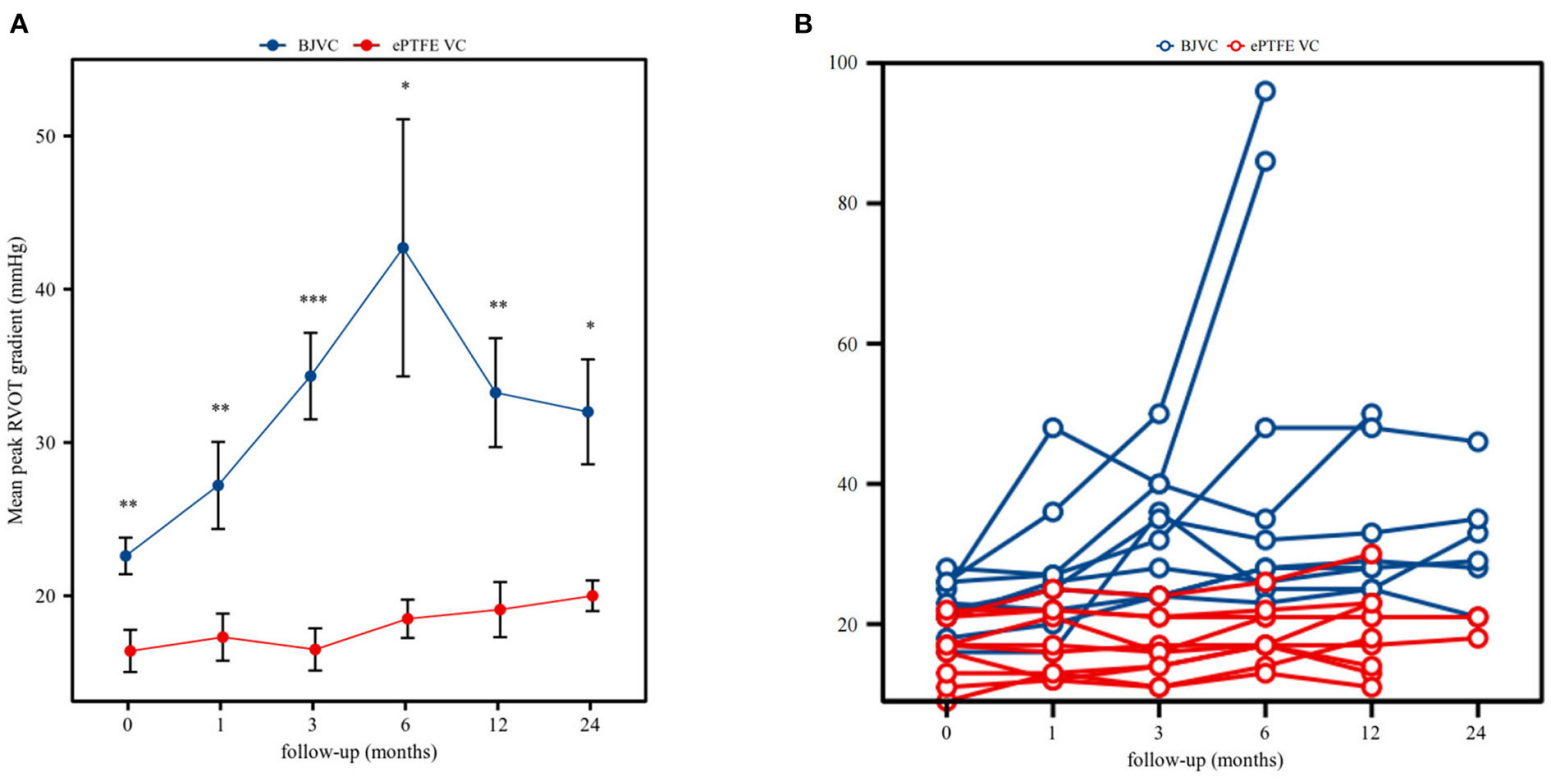
**(A,B)** all show the Peak RVOT gradient dynamics in this cohort during the two year follow-up.

**Figure 4:**
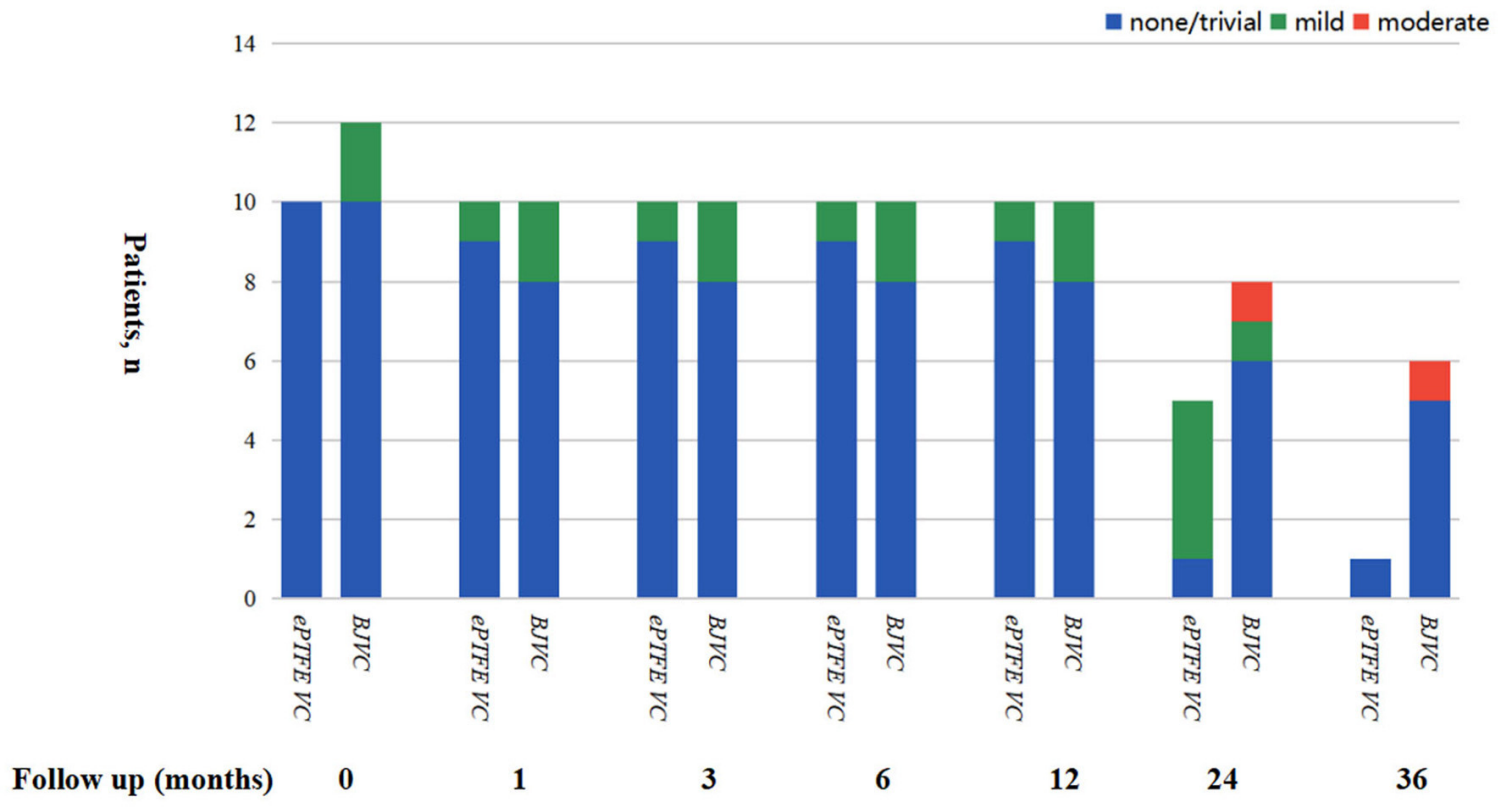
Right ventricular outflow tract regurgitation rate in this cohort during follow-up.

The mean follow-up time of the DP-BJVC group was 29.75 ± 18.31 months, whereas that in the ePTFE VC group was 20.10 ± 6.65 months, with no significant difference (p = 0.111). The survival rate at 4 years was 83.3% in the DP-BJVC group, whereas that at 3 years in the ePTFE VC group was 100%. There was no difference in survival curves with a *P*-value of 0.186 between the two groups (Figure 2A). A higher peak gradient of RVOT of 22.6 ± 3.78 mmHg was found in the DP-BJVC group than those in the ePTFE VC group of 16.4 ± 4.35 mmHg immediately after surgery. The transprosthetic gradient gradually increased with time in both groups, which was significantly higher in the DP-BJVC group at follow-up (*P* < 0.05). Three patients with a RVOT peak gradient exceeding 50 mmHg were found in the DP-BJVC group, and the highest RVOT peak gradient of 42.7 ± 26.52 mmHg was found in the DP-BJVC group at 6 months (Figure 3). There was no severe insufficiency found in both groups, but there were two patients with moderate conduit regurgitation in the DP-BJVC group at follow-up (Figure 4). There were no significant differences between Kaplan–Meier freedom from RVOT graft dysfunction curve (*P* = 0.131) (Figure 2B), which was 66.7% in DP-BJVC group at 4 years; whereas that in the ePTFE VC group was 100%.

Two patients in the DP-BJVC group underwent reintervention due to severe xenograft stenosis at 6 months, while there were no reoperations in the ePTFE VC group. The rate of freedom from RVOT reintervention at 4 years was 80% in the DP-BJVC group, and no statistically significant differences in freedom from reintervention curves were found between the two groups, with a *P*-value of 0.146 (Figure 2C).

## Discussion

Both conduits showed excellent early and midterm outcomes for RVOT reconstruction in Ross procedures. We suggest that DP-BJVC is more suitable for infants, and the ePTFE conduit is more suitable for older children who require larger valve conduits.

The Ross procedure was first introduced by Ross in 1967 (12) and the updated registry results showed favorable outcomes regarding increased follow-up time. The survival rate improved unexpectedly. Reoperation was not as infrequent as anticipated and often involved multiple valves in addition to the coronaries and ascending aorta (13, 14). The conduit in the RVOT and pediatric patients were considered to be risk factors for reoperation (15). In this study, we summarized the result of 22 Ross pediatric patients using DP-BJVC and ePTFE VC for RVOT reconstruction and found that there were no difference in the rate of survival, freedom from RVOT graft dysfunction, and reintervention in the follow-up time between the two groups.

Early mortality in our cohort was 9.1%, which is higher compared to previous reports that demonstrate an operative mortality rate of 3.2% (range: 0.3–6.8%) (16). The two patients who died were in the DP-BJVC group. The present study confirms that patient age, previous cardiovascular surgery, urgency of the procedure, preoperative hemodynamic status, and disease cause, such as infective endocarditis, may carry formidable hospital risk (17). Luciani et al. (18) found that hospital survival may be influenced by center experience, including greater familiarity with complex left ventricular outflow tract anatomy and coronary translocation, and maintenance of proficiency with the Ross procedure in pediatric cardiovascular surgery units. This only reflects the developing learning curve of the Ross procedure in our center, and there is no link to the type of conduits used in RVOT reconstruction.

Previously, cryopreserved homograft conduits showed excellent performance in patients with RVOT reconstruction, especially in adult Ross procedure patients (19). However, inadequate durability and a severe shortage of small sizes of homograft limited the use in infants and young children, and alternative valve conduits needed to be urgently explored

to reconstruct the RVOT (20). The Contegra^®^ conduit, as an alternative, showed comparable or even better performance than homograft (21). In contrast, an unacceptably high rate of dysfunction due to rapid calcium degeneration was found in glutaraldehyde-treated pericardial xenografts. Therefore, epoxide-treated xenografts were suggested by Sharifulin et al. due to excellent performance when compared to allografts, with a rate of freedom from reoperation of 98.8% during long-term follow-up (1). Since 2002, our team have been engaged in DP-BJVC research which clearly elucidates favorable biocompatibility, tissue structure stability, and greater calcification resistance compared to glutaraldehyde-treated BJVC in a series of animal experiments in rats or dogs (22, 23). A satisfactory freedom from first reintervention of 83.4 and 67.3% at 5 and 10 years, respectively, and appropriate dilation with age, was found in children with congenital heart defects after applying the DP-BJVC for RVOT reconstruction in our previous study (8). In the present study, a similar result of freedom from reintervention (80%) was found in the DP-BJVC group at the four-year follow-up. We believe that this conduit could be considered an alternative to homograft.

Although DP-BJVC has offered superior pliability and good handling characteristics in previous pediatric patients with RVOT reconstruction, the availability of larger sizes of this conduit material has been limited in our center, especially for diameters over 18 mm. Since Yamagishi first used thick ePTFE membrane as the pulmonary valve material to reconstruct RVOT of the Tetralogy of Fallot in the 1990s, modified hand-made ePTFE valved conduits have been developed consisting of three bulging sinuses and a tricuspid fan-shaped valve in Japan (24, 25). Long-term follow-up results show comparable durability to the homograft and BJVC, and they appear superior to bioprosthetic valves (26). Shinkawa et al. also reported a significant difference in freedom of reintervention among conduit materials with a diameter ≥18 mm: 88.3; 97.3; 94.2; 100; and 87.5% at 5 years for pulmonary homograft, valved ePTFE conduit, Hancock conduit, non-valved ePTFE tube, and others, respectively (27). In China, Bin Jia et al. found similar results of hand hand-sewn trileaflet valved conduits, with lower incidences of graft failure than conventional BJV grafts at a median follow-up of 16.5 months (range 1–48 months) (28). In our cohort, the freedom of reintervention of ePTFE VC of 100% seems to be superior to that of DP-BJVC, although there is no statistically significant difference between the two groups. Besides, the transprosthetic gradient was significantly lower (16.4 ± 4.35 mmHg) and only mild conduit regurgitation was found in the ePTFE group. Contrastingly, the highest RVOT peak gradient (42.7 ± 26.52 mmHg) was found at 6 months, and two patients with moderate regurgitation were found in the DP-BJVC group, although no statistically significant difference was found regarding graft dysfunction. We believe that the smaller diameter of the DP-BJVC than that in the ePTFE group may have resulted in the higher RVOT peak gradient, although no difference in z-score was found between the two groups. Additionally, the two patients with moderate regurgitation in the DP-BJVC group may have been as a result of an abnormality of the left side of the heart; one patient with MVR and the other with coarctation repair. In addition, although our method of sewing trileaflet valved conduits is simpler than that of Yamagishi, and unlike that of Bin Jia et al. who require special instruments, our recent outcomes are not significantly different from those studies in terms of durability. We believe our simplified suture method is easier and may gain popularity in other regions and countries.

Unlike adult patients, children cannot avoid reoperation due to their somatic growth. Small graft size is one of the most frequently cited critical factors limiting conduit longevity. Yamashita et al. (29) show that a smaller-sized conduit is strongly related to conduit stenosis, and propose that the first choice of conduit for RVOT reconstruction be an ePTFE conduit with a z-score of approximately 1.4 in infants and younger children. In our cohort, the DP-BJVC group patients had different characteristics including younger age, lighter weight, and smaller diameter than the ePTFE VC group. Our previous study found that the DP-BJVC conduit performed satisfactorily in terms of functionality and durability, especially for patients ≤ 3 years old, due to the ability to appropriately dilate with age (8). We, therefore, suggest that DP-BJVC is more suitable for younger children.

There are several limitations of this study. Firstly, this study was a non-randomized, observational, retrospective review. Secondly, a relatively small cohort of patients was enrolled and may result in the the bias that the higher RVOT peak gradient was found in the DP-BJVC group with a smaller diameter than that in the ePTFE. We will need to strengthen our results through future larger studies. Additionally, it may take longer to verify the clinical outcomes of the double-valve conduit, especially for the ePTFE VC group with a mean follow-up time of only 20 months. More participants and a longer follow-up time are also needed to identify the risk factors of conduit dysfunction.

## Conclusion

Both DP-BJVC and ePTFE VC show excellent early and midterm outcomes for RVOT reconstruction in the Ross procedure. The ePTFE VC had improved function compared to the DP-BJVC, especially in terms of conduit regurgitation and RVOT peak gradient. Considering the ability to appropriately dilate with age accordingly, we suggest that DP-BJVC is more suitable for infants, and the ePTFE conduit is more suitable for older children who require larger conduits.

## Data availability statement

The original contributions presented in the study are included in the article/Supplementary material, further inquiries can be directed to the corresponding author.

## Ethics statement

The studies involving human participants were reviewed and approved by the Institutional Ethics Committee (Reference Number: LYF2020097). Written informed consent to participate in this study was provided by the participants' legal guardian/next of kin.

## Author contributions

CH, ZW, and HY developed the idea of the study, participated in its design and coordination, and helped to draft the manuscript. TL, HZ, TQ, and SW contributed to the acquisition and interpretation of data. JC, QW, and YY provided critical review and substantially revised the manuscript. All authors read and approved the final manuscript.

## Funding

This work was supported by the Major Scientific and Technological Projects for collaborative prevention and control of birth defects in Hunan Province (2019SK1010) and Hunan Provincial Natural Science Foundation of China (Grant Number: 2020JJ4787).

## Conflict of interest

The authors declare that the research was conducted in the absence of any commercial or financial relationships that could be construed as a potential conflict of interest.

## Publisher's note

All claims expressed in this article are solely those of the authors and do not necessarily represent those of their affiliated organizations, or those of the publisher, the editors and the reviewers. Any product that may be evaluated in this article, or claim that may be made by its manufacturer, is not guaranteed or endorsed by the publisher.

## Supplementary material

Supplementary Video 1 Hand-Sewn ePTFE Valved Conduit preparation.
